# Regulation of expression of human RNA polymerase II-transcribed snRNA genes

**DOI:** 10.1098/rsob.170073

**Published:** 2017-06-14

**Authors:** Joana Guiro, Shona Murphy

**Affiliations:** Sir William Dunn School of Pathology, University of Oxford, Oxford, UK

**Keywords:** snRNA, transcription, RNA processing, Integrator, Mediator

## Abstract

In addition to protein-coding genes, RNA polymerase II (pol II) transcribes numerous genes for non-coding RNAs, including the small-nuclear (sn)RNA genes. snRNAs are an important class of non-coding RNAs, several of which are involved in pre-mRNA splicing. The molecular mechanisms underlying expression of human pol II-transcribed snRNA genes are less well characterized than for protein-coding genes and there are important differences in expression of these two gene types. Here, we review the DNA features and proteins required for efficient transcription of snRNA genes and co-transcriptional 3′ end formation of the transcripts.

## 1. Introduction

RNA polymerase II (pol II) is responsible for the transcription of all 25 000 or so protein-coding genes in the human genome. Many non-coding RNAs, including ribosomal (r)RNA, transfer (t)RNA, the 7SK RNA that regulates transcription elongation by pol II, and the spliceosomal U6 and U6atac small nuclear (sn)RNAs are transcribed by pol I or pol III [1–4]. However, the non-coding microRNAs, small nucleolar (sno)RNAs and the remaining snRNAs are transcribed by pol II [2,5,6]. SnRNAs are short RNAs (less than 350 nts) that associate with proteins to form small nuclear ribonucleoprotein particles (snRNPs) [6].

Due to their roles in pre-mRNA splicing, the pol II-transcribed U1, U2, U4, U4atac, U5, U11 and U12 snRNAs are required for expression of intron-containing protein-coding genes. The major spliceosome includes the U1, U2, U4, U5 and U6 snRNPs and recognizes the canonical GT/AG splice sites flanking introns to assemble in a stepwise manner with the pre-mRNA. Some introns are instead flanked by AT/AC splice sites, which recruit the minor spliceosome containing instead U11, U12, U4atac, U5 and U6atac snRNPs [7]. U7 snRNA is required for 3′ end formation of replication-activated histone mRNA [8] and the U3 sn(o)RNA is required for processing of rRNA [9].

## 2. DNA sequence elements involved in expression of pol II-transcribed snRNA genes

**Figure 1.**
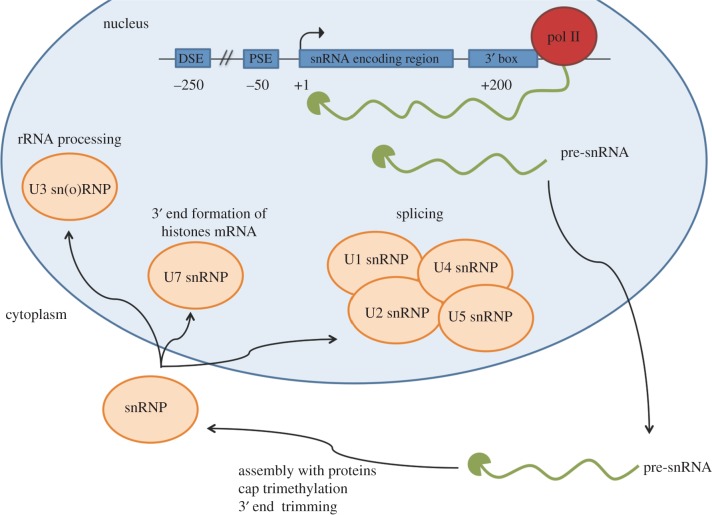
Expression of human snRNA genes. Distal sequence element (DSE) and proximal sequence element (PSE) are the enhancer and promoter elements, respectively. The 3′ box is the snRNA gene-specific RNA processing element. An arrow represents the start site of transcription and the numbers below the line indicate the approximate position of the elements with respect to the transcription start site. DSE comprises the Oct1 binding site ATTTGCAT [12], three Sp1 binding sites (G/T)(G/A)GGCG(G/T)(G/A)(G/A)(G/T) [13] and a STAF binding site YY(A/T)CCC(A/G)N(A/C)AT(G/C)C(A/C)YYRCR [14]. The PSE sequence consensus sequence is TCACCNTNA(G/C)NNNAA(A/T)(G/A)N [2]. The consensus sequence of the 3′ box is GTTYN_0-3_AARRYAGA [15]. The snRNA transcript is represented in green with the cap in the 5′ end. The pre-snRNA is exported to the cytoplasm for maturation by 3′ end trimming, cap trimethylation and assembly with the snRNP proteins [6]. The functions of the various snRNPs after reimport into the nucleus are noted.

Although the functions of snRNAs are well understood, the regulation of their expression is still not fully characterized. The human snRNA genes have a much simpler promoter structure than most protein-coding genes; this comprises a distal sequence element (DSE) which acts as an enhancer and an essential snRNA gene-specific proximal sequence element (PSE) which is the core promoter [2,10,11] (figure 1). The transcripts are intronless and non-polyadenylated, and a 3′ box directs formation of the 3′ end of the pre-snRNA, which is further processed to produce the mature snRNA [10].

In vertebrates the genes for the major snRNAs (U1, U2, U4 and U5) are well conserved with recognizable DSE, PSE and 3′ box sequences [2], and *Drosophila* snRNA genes also have PSE-like sequences [16]. The vertebrate snRNA genes are often present in multiple copies. For example, there are four copies of the U1 snRNA gene/human haploid genome) [17] and 15 copies of the U2 snRNA gene/human haploid genome) [18]. In contrast, the genes for the minor snRNAs (U11, U12 and U4atac) are usually single-copy genes and are missing in many invertebrates including Cnidaria and Annelida [19].

An important feature of snRNA genes is the obligatory coupling between the 3′ box RNA processing element and the snRNA gene-type promoter; replacement of an snRNA gene promoter with the promoter of a protein-coding gene results in failure of RNA 3′ end formation [15,20].

The pol III-transcribed genes for 7SK snRNA and U6 snRNA also have a DSE and PSE, but in addition they have a TATA box at −25 [2]. Placing a TATA box downstream of the PSE in a pol II-transcribed snRNA gene converts transcription from pol II to pol III [2], emphasizing that the DSE and PSE can work as cis-acting elements for either polymerase.

Here, we review what is currently known about expression of human snRNA genes transcribed by pol II and the future prospects of a complete understanding of the mechanisms involved.

## 3. Transcription factors associated with snRNA genes

**Figure 2.**
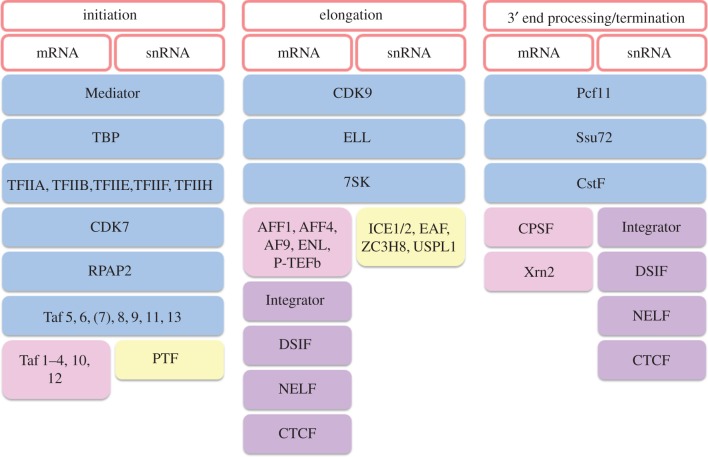
Factors regulating expression of snRNA and protein-coding genes. In blue are the factors that have roles in the same event of transcription in both classes of genes. In pink and yellow are the factors unique to protein-coding genes and snRNA genes, respectively. Factors that regulate both genes but at different steps are shown in purple [22,24,26–55].

Transcription factors Oct1, Sp1, NF1 and Staf bind to sequences in the DSE, which has the properties of a transcriptional enhancer [2,21]. Oct1 enhances snRNA gene expression by stabilizing binding of the PSE-binding protein/PSE-binding transcription factor/snRNA activating protein complex, PTF (also known as PBP and SNAPc) [12,22,23], to the PSE of both pol II- and pol II-transcribed snRNA genes through direct interaction [12,24,25]. In turn, PTF helps to recruit the TATA-binding protein (TBP) and the TBP-associated factors (TAFs) that make up the snRNA TAF complex (snTAFc) to the pol II-transcribed snRNA genes [22,26,27]. The snTAFc on the U2 snRNA gene, which comprises TAF5, TAF6, TAF8, TAF9, TAF11 and TAF13 [27], is a subset of the TAFs found in TFIID, the TBP/TAF-containing complex required for transcription of many protein-coding genes [28]. However, the U1, U4, U5 and U11 snRNA genes appear to have a different TAF complex, which includes TAF7 [29]. TAF7 may help to recruit the large multisubunit Mediator complex, which is required for efficient transcription of snRNA genes [29]. TAF7 has also been implicated in regulation of promoter escape (see below). However, TAF7 appears to be absent from the U2 snRNA genes [27], suggesting that these genes employ a different mechanism of Mediator recruitment and promoter escape. The general protein-coding gene transcription factors TFIIA, TFIIB, TFIIE, TFIIF and TFIIH are also required for transcription of snRNA genes [30,31]. Figure 2 indicates which factors are shared between snRNA genes and protein-coding genes and which are specific to each class of gene. As noted above, the DSE and PSE are interchangeable between the pol II- and pol III-transcribed snRNA genes [2]. However, in the presence of a TATA box in the promoter, a pol III- and snRNA gene-specific TBP-containing complex is recruited [2].

There is evidence that a nucleosome between the DSE and PSE in the U1, U2, 7SK and U6 snRNA genes brings those two regions together, facilitating the interaction between Oct1 and PTF [56–59]. In addition, PTF plays a role in depletion of histones from the transcription unit of the U2 gene [32]. Thus, chromatin structure plays a role in the regulation of expression of human snRNA genes. Expression of the pol III-transcribed U6 snRNA gene is regulated by interaction of a protein involved in chromatin modification, the chromodomain-helicase-DNA binding protein 8 (CHD8), with Staf [60]. As CHD8 has also been shown to interact with the elongating form of pol II [60], it may also regulate transcription of pol II-transcribed snRNA genes.

## 4. Pol II carboxyl terminal domain phosphorylation and snRNA gene expression

The largest subunit of mammalian pol II has a carboxyl terminal domain (CTD) comprising 52 repeats of the consensus heptapeptide Y_1_S_2_P_3_T_4_S_5_P_6_S_7_. During transcription, the phosphorylation of the CTD helps to recruit transcription and RNA processing factors at the right point of the transcription cycle [61].

**Figure 3.**
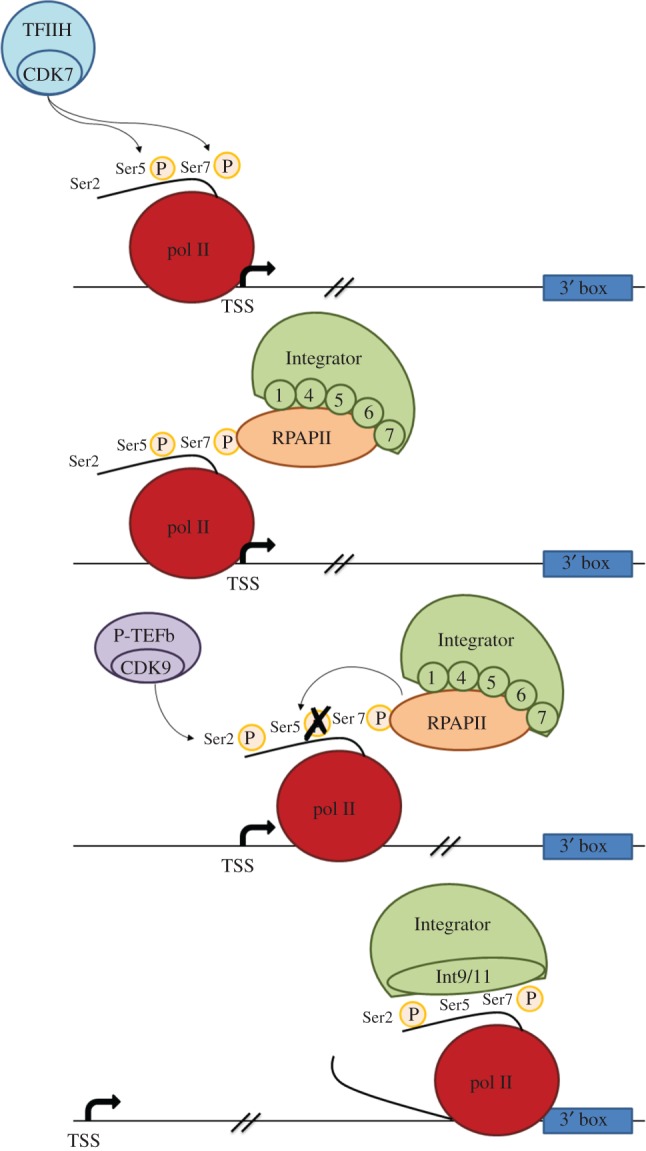
Pol II CTD phosphorylation events in snRNA gene transcription. Initially, the cyclin-dependent kinase (CDK)7 subunit of TFIIH phosphorylates Ser5 and Ser7. RPAP2 interacts with Ser7P. RPAP2 in turn recruits the Integrator subunits Int1, Int4, Int5, Int6 and Int7. RPAP2 dephosphorylates Ser5P, and positive-transcription elongation factor b (P-TEFb) is recruited by a mechanism still unknown. The P-TEFb subunit CDK9 phosphorylates Ser2. The double phosphorylation on Ser2 and Ser7 recruits Int9/11, which allows RNA processing to occur [62].

Ser7 is phosphorylated during transcription of all human genes. However its phosphorylation appears to be essential only for expression of snRNA genes [33]. Ser5 and Ser7 are both phosphorylated by the cyclin-dependent kinase 7 (CDK7) subunit of TFIIH soon after initiation of transcription [34] and phospho-Ser7 (Ser7P) mediates recruitment of the phospho-Ser5 (Ser5P) phosphatase RPAP2 [62]. RPAP2 not only dephosphorylates Ser5P soon after initiation of transcription but also helps to recruit subunits Int1, Int4, Int5, Int6 and Int7 of the large multisubunit Integrator complex [62]. The complete Integrator complex comprises 14 subunits and is responsible for the recognition of the 3′ box and RNA cleavage that produces pre-snRNA [35]. However, the catalytic subunit Int11 is absent when Integrator is associated with RPAP2 [62], and Int9 is also likely to be absent as it interacts strongly with Int11 [63]. The CDK9 kinase subunit of the positive-transcription elongation factor b (P-TEFb), which is involved in the expression of both protein-coding genes and of snRNA genes, phosphorylates Ser2 (Ser2P) of the pol II CTD [61] and Ser7 and Ser2 phosphorylation together recruit the missing subunits Int9/11 to activate 3′ box recognition and RNA 3′ end processing [64]. Accordingly, CDK9 inhibitors have a drastic effect on 3′ end processing of snRNA gene transcripts [36]. This sequence of events is shown in figure 3.

Ser2 can also be phosphorylated by CDK12 [65,66] and CDK12 knockdown affects snRNA gene expression [67]. Phosphorylation of the DRB-sensitivity-inducing factor (DSIF) and the negative elongation factor (NELF) by P-TEFb are also required for productive elongation of protein-coding genes [37,38,68]. However, CDK9 inhibitors have little effect on transcription of the U1 and U2 genes [39].

After initiation of transcription of protein-coding genes, the capping machinery is recruited to the 5′ end of the nascent transcript and allosterically activated by Ser5P [69]. As snRNA gene transcripts are also co-transcriptionally capped [70], it is likely that Ser5P also ensures capping of pre-snRNAs.

## 5. The role of Mediator in expression of snRNA genes

Association of the large 26-subunit Mediator complex with protein-coding genes has been well studied. This complex is recruited to the pre-initiation complex (PIC) and can bind to several transcription factors at the same time. Mediator has an important role as a binding platform for interaction between the transcription factors bound to sequences in protein-coding gene promoters and pol II [40].

The Mediator complex is made up of modules, with the head module comprising the subunits Med6, Med8, Med11, Med17, Med18, Med20 and Med22 [71]. The head module is responsible for the interaction with the pol II CTD through Med17 [72]. Med17 is a subunit on the ‘nose’ of the head module whereas Med18 and Med20 are located on the ‘jaw’. A mutation on the alpha helix of Med18 is predicted to strengthen the interaction between the jaw and the rest of the module. The jaw is therefore thought to be the moveable element that regulates binding to the pol II CTD [71]. This flexibility may also have a role in interactions between Mediator and pol II and also between Mediator and transcription factors such as TFIIH [73] and TBP [74].

The association of Mediator subunits with snRNA genes was only described for the first time recently. Med1, Med23 and Med26 are associated with a subset of snRNA genes (U1, U4 and U5) and in significantly higher levels than in the promoter region of some protein-coding genes [29]. However, the ChIP experiments did not define the region of the snRNA genes associated with Mediator. Med26 is required for the expression of U1, U2, U4, U5 and U11 genes [29] and is essential for the recruitment of the little elongation complex (LEC) [29]. Interestingly, Med26 also interacts with TAF7, which represses initiation and blocks LEC recruitment. In addition, TAF7 has protein sequence similarities to EAF, which is part of the LEC. Accordingly, it has been suggested that EAF could snatch Med26 from TAF7 to facilitate elongation [29]. Med26 may therefore act as a molecular switch triggering elongation after promoter escape, at least for the snRNA genes where TAF7 is recruited.

## 6. The little elongation complex and elongation of transcription

Gene expression is regulated not only at transcription initiation, but also during elongation. Pol II pauses soon after initiation of transcription of protein-coding genes at an early elongation checkpoint (EEC) due to the negative elongation factors, NELF and DSIF. Phosphorylation of the pol II CTD, NELF and DSIF by the CDK9 subunit of P-TEFb releases pol II from the EEC to allow productive elongation [68]. A complex named the super elongation complex (SEC) comprising the elongation factor ELL, AFF1, AFF4, AF9, ENL and P-TEFb is involved in productive elongation of transcription of protein-coding genes [41].

Instead of the SEC, another ELL-containing complex specifically regulates elongation of transcription of pol II-transcribed snRNA genes. This LEC comprises ELL, ICE1, ECE2, EAF and ZC3H8 [42]. It has been suggested that ICE1, ICE2, ELL and EAF are recruited to the promoter by the Mediator complex [29]. ICE1 has been shown to be the scaffold for LEC formation and is essential for recruitment of the other components. ELL interacts with the ICE1 N-terminal domain, whereas ICE2 and ZC3H8 interact with the ICE1 C-terminal domain. In addition, ICE1 is required for pol II recruitment [42]. ELL knockdown shifts the distribution of pol II on snRNA genes towards the 5′ end, as expected of a factor that facilitates transcription elongation [75]. It has been suggested that ELL is necessary to ensure a high level of transcription of snRNA genes [75]. The function of subunits ICE2 and ZC3H8 is currently unknown.

The LEC has been shown to co-localize with coilin [42], which is found in Cajal bodies, where small nuclear ribonucleoprotein (snRNP) maturation takes place [6]. ICE1 recruits another protein, USPL1, to Cajal bodies. USPL1 also interacts with U1 and U2 snRNPs and its knockdown causes a reduction in snRNA levels [43]. However, the molecular mechanism underlying the effect of USPL1 knockdown is unclear.

More recently, it has been shown that the 7SK snRNP, comprising 7SK RNA, MePCE and Larp7, plays a role in the recruitment of the LEC to snRNA genes. Knockdown of 7SK RNP components disrupts LEC integrity, affects pol II recruitment and reduces expression of snRNA genes [76]. This was a surprising finding as the 7SK RNP is a negative regulator of the CTD Ser2 kinase P-TEFb complex [68].

## 7. The Integrator complex and snRNA genes

The Integrator complex was discovered more recently than Mediator. It is present only in metazoans and is a large complex of 14 subunits and is required for 3′ end processing of pol II-transcribed pre-snRNAs in humans and flies [33,35,44].

Integrator is able to bind the pol II CTD phosphorylated on both Ser2 and Ser7 [64]. However, it is still unclear which subunits are involved in Ser2P and Ser7P recognition. Int11 is a paralogue of CPSF-73, which is responsible for RNA cleavage directed by poly(A) sites and the RNA processing signal in the replication-activated histone pre-mRNAs [77]. Depletion of Int11 causes accumulation of the pre-U1 and pre-U2 snRNAs [32,35], which is in agreement with the expected catalytic role of this subunit.

So far, Integrator subunits have not been found to interact directly with chromatin. However, subunit Int12 has a PHD (plant homeodomain) domain, which is a common chromatin-binding motif that recognizes methylated histones [78]. Subunits Int3 and Int6 are also part of another complex involved in detecting DNA double strand breaks, called sensor of single stranded DNA (SOSS) [79–83]. As snRNA genes are highly expressed and at least the U2 snRNA gene is depleted of canonical histones [45], it has been suggested that these Integrator subunits could help to prevent DNA damage that is facilitated by a constitutively open chromatin state [84].

Int5 is found associated with both the snRNA gene promoter region and the 3′ box, whereas Int11 is associated mainly with the 3′ box [62]. Integrator may therefore be a modular complex that assembles in a stepwise manner, with some subunits playing a role early in transcription, while others are active in RNA 3′ end processing.

It has been demonstrated that Int4 interacts with NELF-A and Int6 interacts with the Spt5 subunit of DSIF [46]. In expression of snRNAs genes, DSIF has been proposed to bind pol II prior to transcription elongation [46]. Integrator could therefore be recruited both by pol II CTD phosphorylation and by association with DSIF. Recruitment of NELF through interaction with RNA and Integrator could contribute to specific 3′ box recognition by preventing recruitment of the cleavage stimulatory factor (CstF) polyadenylation factor [46]. These events would then allow Integrator to properly process the nascent snRNA transcript [46].

Until recently Integrator was thought to be an snRNA-specific processing complex, but it has now been shown to also regulate expression of protein-coding genes [47–49]. Expression of the immediate early genes (IEGs) activated by epidermal growth factor (EGF) is regulated by pausing prior to elongation and this pause is overcome by EGF induction. EGF induction regulates the recruitment of Integrator, which in turn recruits the SEC [47]. Integrator also plays a role in release of the NELF-mediated pause during transcription of human genes and the HIV genome [48,49]. Interestingly, the human genes targeted by Integrator possess 3′ box-like sequences close to the end of the genes, and show a decrease in transcript levels upon Int11 knockdown, suggesting that Int11 is also involved in the production of mRNA [48]. Integrator therefore appears to play major roles in expression of protein-coding genes.

## 8. The 3′ box and termination of transcription of snRNA genes

The 3′ box is a signal for processing nascent snRNA transcripts and pol II continues transcribing for a few hundred base pairs past the 3′ box before transcription is terminated [39]. As already mentioned, Integrator binds DSIF, NELF and pol II, recognizes the 3′ box signal on the RNA and processes the nascent transcript. Pre-snRNA 3′ end formation and pol II transcription termination on snRNA genes is intrinsically linked, as demonstrated by a recent study in which knockdown of the catalytic subunits Int9 and Int11 caused termination defects [32]. NELF is also involved in termination. It accumulates downstream of the 3′ box of the U2 snRNA gene and its knockdown also causes a termination defect [45]. A CTCF binding site is located close to the region of NELF association, and delimits a nucleosome-depleted region spanning the transcription unit [45]. CTCF is best known as a transcription factor for protein-coding genes through its action as an insulator and organizer of chromatin structure [85]. CTCF has been shown to position nucleosomes and to impede pol II elongation [50,86]. By acting in this way, CTCF could control the chromatin structure and elicit termination of transcription. U2 snRNA genes may therefore have a ‘late’ elongation checkpoint where CTCF-positioned nucleosomes slow down pol II, allowing NELF to terminate transcription [45].

**Figure 4.**
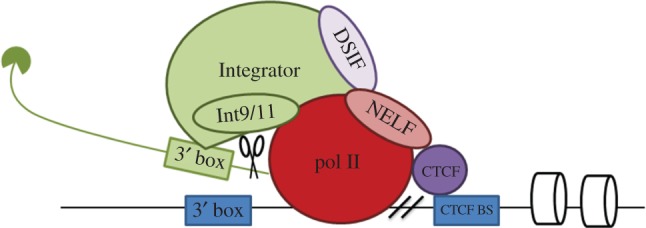
Model for transcription termination of the U2 snRNA gene. The snRNA transcript is represented in green with the cap in the 5′ end and nucleosomes are represented by barrels. Pol II continues to transcribe after the 3′ box and Integrator processes the nascent RNA after 3′ box recognition. CTCF recognizes the CTCF binding site downstream of the 3′ box and controls nucleosome occupancy. Negative elongation factor (NELF) is recruited by DRB-sensitivity-inducing factor (DSIF) and CTCF at the end of the transcription unit and causes transcription termination [51].

In agreement with these findings, a more recent study implicates CTCF in DSIF, NELF and P-TEFb recruitment to the U2 snRNA gene [51]. The findings suggest a model linking transcription termination to recognition of the 3′ box. In this model, NELF is required for termination of transcription, since its knockdown results in termination defects without affecting CTCF recruitment or RNA 3′ end formation. Additionally, P-TEFb phosphorylates the pol II CTD on Ser2, leading to the recruitment of Integrator subunits 9/11, which can elicit RNA processing driven by the 3′ box [51]. This model would explain how transcription termination and RNA processing are tightly linked during expression of snRNA genes (figure 4).

Intriguingly, mRNA 3′ end processing factors Pcf11, Ssu72 and Cstf64 also associate with snRNAs genes [32]. However, Pcf11 and Ssu72 were shown to affect termination of transcription of snRNA genes rather than 3′ end processing.

## 9. Coupling of initiation and RNA 3′ end formation

The 3′ box is only recognized if transcription is initiated from a pol II-dependent snRNA gene promoter [15,20]. If the PSE is substituted by a protein-coding gene promoter, the transcripts become longer as the 3′ box signal is ignored and pol II transcribes until a functional polyadenylation site is reached [87]. The mechanism underlying coupling of transcription initiation and RNA 3′ end formation is still not understood but may involve the snRNA gene-specific promoter factor PTF or the snRNA genes-specific components of the LEC. Alternatively, recognition of the 3′ box may only occur in the absence of SEC recruitment.

## 10. snRNA pseudogenes and variant snRNA genes

With the exception of teleosts and birds, all metazoans studied so far have pseudogenes derived from snRNA genes, with mammals possessing a large number [19]. Some sequences in the human genome that were previously thought to be U1 snRNA pseudogenes have been shown to be true genes that have recognizable DSE, PSE and 3′ box sequences and are actively transcribed [17]. These genes have been termed variant (v)U1 genes [17,88]. The DSE, PSE and 3′ box sequences of the vU1 snRNA genes are, however, sufficiently different from the U1 snRNA gene sequences to suggest that they are differentially regulated. Interestingly, the vU1 snRNAs appear to be most highly expressed in embryonic stem cells [17]. U1b is a mouse U1 variant gene only expressed in early embryonic stages, although the U1 and U1b snRNAs differ by only seven base changes [89,90]. However, the promoter of U1b differs significantly from that of U1 [90]. Although the function of U1b is still unclear, there are clear parallels between mouse U1b and the human vU1 snRNAs.

U5 snRNA variants in human and *Drosophila* have also been described [91,92]. The variants form different functional snRNP complexes from the canonical U5 snRNP and are thought to play roles in splicing [91]. Interestingly, the U5 variants are differentially expressed during development and may promote tissue-specific splicing [92].

## 11. Conclusion

The role of general transcription factors, CTD phosphorylation and the Integrator complex in expression of snRNA genes has been well characterized in the last two decades. The recent elucidation of the roles of DSIF, CTCF and NELF in expression of snRNA genes suggests how 3′ end processing of snRNA transcripts is linked to transcription termination. Also, it has been shown that mRNA 3′ end processing factors such as Pcf11 and Ssu72 are involved in termination of transcription of snRNA genes. We can now appreciate that there are many transcription factors shared by protein-coding genes and snRNA genes, although some play distinct roles in expression of the two gene types (figure 2).

Despite all this new information, there are still some important aspects of the expression of pol II-transcribed snRNA genes that remain to be elucidated. For example, how is initiation of transcription of these genes so tightly coupled to 3′ end formation of the transcripts? Why would U2 snRNA gene expression be regulated by different mechanisms than the U1, U4 and U5 snRNA genes? Why do snRNA genes require a specific elongation complex that is recruited by a non-coding RNA? Does the Mediator complex on the snRNA genes differ from that on protein-coding genes? And why does the herpesevirus transactivator protein VP16 only activate transcription of protein-coding genes and not snRNA genes [93] when activation occurs through interaction with TAF9 [94] and TFIIA [95]? However, VP16 also interacts with Med25 [96] and it is currently unclear whether this is recruited to snRNA genes.

Hopefully, future research will provide the answers necessary for a full understanding of the regulation of expression of this important class of pol II-transcribed genes.
